# Production and properties of particleboard and paper from waste poppy straw

**DOI:** 10.1038/s41598-024-82733-9

**Published:** 2025-01-02

**Authors:** Kateřina Hájková, Tomáš Holeček, Michaela Filipi, Josef Bárta, Adam Sikora, Uğur Özkan

**Affiliations:** 1 Faculty of Forestry and Wood Sciences, Czech University of Life Science Prague, Kamýcká 129, Prague, 165 00 Czech Republic; 2 Faculty of Chemical Technology, Institute of Chemistry and Technology of Macromolecular Materials, University of Pardubice, Studentská 572, Pardubice, 532 10 Czech Republic; 3 Faculty of Forestry, Isparta university of Applied Science, Isparta Merkez/Isparta, Çünür, 32260 Turkey

**Keywords:** Poppy straw, Soda pulp, Nitrate-alkaline pulp, Particleboard, Mechanical properties, Biomaterials, Biochemistry, Materials chemistry, Polymer chemistry, Surface chemistry

## Abstract

Due to the scarcity of wood in some countries, it is necessary to replace it with other raw materials and at the same time use the waste material. The aim of this research is to use poppy waste straw for the efficient conversion of possible lignocellulosic materials – pulps and particleboards. Their suitability for the production of composites is assessed on the basis of selected physical or mechanical properties. Tensile strength index, burst strength index and air permeability by Gurley have been identified as critical properties of pulp made from poppy straw through two delignification methods. The better mechanical properties, i.e., tensile strength index, were achieved at 52.7 N·m/g for the sodium pulp, but the nitrate-alkali method also showed corresponding values at 45.9 N·m/g. Similar parameters to those of bagasse or similar fast-growing plants were achieved in particleboard production. The results of this research are used to evaluate poppy straw as an alternative raw material to produce biocomposites.

## Introduction

The importance of sustainability and environmental preservation has seen a notable rise in the modern era. Waste management and adopting renewable energy sources have emerged as pivotal strategies to mitigate environmental harm and optimize resource us-age. Consequently, biomass has emerged as a sustainable, abundant, and eco-friendly renewable energy source. Due to these advantages, there has been growing interest in using biomass for energy generation. Significant quantities of agricultural and forestry residues are generated, so they represent a promising resource for obtaining valuable outputs. These materials can be transformed through various biotechnological processes to yield beneficial biobased chemicals and fuels^1,2^.

Biomass finds diverse applications ranging from animal feed and fertilizer to energy and heat production, particularly sourced from food and agricultural waste. Using ligno-cellulosic waste to generate valuable products signifies a sustainable and environmentally friendly resource potential^3^. The global annual production of lignocellulosic biomass is estimated to reach approximately 181.5 billion tons^4^. Utilizing agricultural residues containing lignocellulosic materials as biomass offers advantages. Such advantages as abundant availability, renewable sourcing, and cost-effective production^5^. Given concerns regarding deforestation for wood supply in the forestry sector, there have been extensive studies exploring alternative non-wood sources for the forest products industry, notably for biocomposites^6–9^and paper production^6^. Consequently, numerous agricultural wastes/residues and naturally grown annual plants worldwide exhibit wood-like chemical and physical properties^10^. These include materials such as poppy (*Papaver somniferum*) stalks^11^, cotton stalks^12^, peanut hulls^13^, sunflower stalks^14^, sugarcane waste^15^, kenaf^16^, vine pruning^17^, coconut husk^18^, and a hybrid of reed fibre/coconut husk particles^19^.

Lignocellulosic materials demonstrate significant potential within the manufacturing industry. They are anticipated to play a pivotal role in the producing composite materials soon, with sustainability being just one aspect of their appeal^20^. There is a growing trend towards using renewable materials in composite production^21,22^. As a result, biomass offers several advantages in particleboard production, including its durable and light properties, making it an attractive alternative.

Using biomass in paper and particleboard manufacturing significantly contributes to conserving natural resources. Nevertheless, it also improves waste management practices and minimizes environmental impacts. Traditionally, paper and particleboards are derived from wood. However, there is a growing interest in utilizing alternative raw materials to reduce environmental footprint and conserve natural resources. Raw materials used in papermaking must contain high cellulose levels, a criterion fulfilled by lignocellulosic biomass comprising cellulose, hemicelluloses, and lignin^23^. A higher cellulose content and lower lignin content in biomass are associated with increased paper durability^24^, thus enhancing service quality^25^.

Poppy (*Papaver somniferum* L.) is an annual herbaceous plant species belonging to the *Papaveraceae*family^26^. The well-known and thoroughly researched *Papaver somniferum* L. is a member of the *Papaver genus*, which has more than 100 species^27^. It has a significant production volume, has been cultivated for various purposes for many years, and is recognized worldwide. Moreover, it usually grows between 60 and 200 cm in height. Poppy originating from the Eastern Mediterranean, has a long history of cultivation in regions such as Anatolia and India^28^.

This study explores the feasibility of using poppy waste biomass for paper with delignification processes (nitrate-alkaline and soda), and particleboard production, with evaluation of its environment and economic impacts.

## Materials and methods

### Materials

The poppy straw (*Papaver somniferum* L.) was grown and harvested from a field in the uplands of the southwestern part of Prague. The bulk density of the poppy straw used was approximately 115 kg/m^3^.

The chemicals for pulp production were nitric acid, sodium hydroxide, and acetic acid. Chemicals for particleboard production - phenol formaldehyde resin. Moreover, chemicals used in chemical analyses include ethanol, toluene (extractives); sulfuric acid (lignin); acetylacetone, 1,4-dioxane, hydrochloric acid, methanol (cellulose); acetone, ethanol, sodium chlorite, acetic acid (holocellulose). All chemicals were supplied by PENTA (Prague, Czech Republic).

### Chemical analysis

Poppy material was grounded using an MF 10 BASIC knife mill from IKA (Staufen, Germany). For chemical analysis, the following methods were performed using Tappi methods: extraction, ash, cellulose, lignin, and holocellulose analysis.

The representation of ash as an inorganic fraction in straw was determined by exposure to 525 °C according to the Tappi T 211 om-02 method^29^. Before further chemical analyses, unwanted extractives were removed. Namely, the sample was extracted in a binary mixture of ethanol-toluene for six hours in Soxhlet apparatus according to the Tappi T 280 wd-06 method^30^. Macromolecular substances were determined as cellulose, lignin, and holocellulose. The analysis of cellulose was carried out using the Seifert method. The isolation of cellulose was done using acetylacetone, dioxane, hydrochloric acid, and methanol^31^. Lignin was determined in the sulphuric acid Klason method according to Tappi T 13 wd-74^32^. The analysis of holocellulose was determined by the chloration method according to Wise^33^, and from holocellulose the representation of hemicelluloses was calculated.

### Production of products from poppy waste

The poppies were first disintegrated into chips, which were used for pulp and particleboard production. Selected properties were determined by the products produced.

### Nitrate-alkaline pulp

In the case of the nitrate-alkaline method, 6% nitric acid was used as the cooking chemical, which nitrated the lignin in our pulp and partially oxidized it to nitro-lignin, which was removed in the second stage of extraction using 5% sodium hydroxide. These two stages took only 45 min, then neutralization with 1% acetic acid and a thorough washing.

### Soda pulp

The poppy chips were placed in a laboratory cooker where the soda cook was run. The cooking chemical used was 19% sodium hydroxide with a hydromodul of 5:1. The process of soda cooking consisted of four phases. In the first phase, the raw material was cooked to 105 °C then impregnated at this temperature for 30 min in the second phase. In the third phase, cooking to 160 °C followed, and at this temperature, the raw material was cooked to an H-factor of 1250 h. The cooking time was 4 h. Subsequently, the pulp was stripped of the black liquor by a four-stage washing process, pulped, separated from rejects, and prepared to produce laboratory handsheets.

### Particleboard

To produce the particleboard, raw materials were prepared using a cutting machine; residues of poppy pods and stalks were cut into a maximum fraction size of 15 mm. The initial raw material had a moisture content of 5%. This prepared raw material was then mixed with phenol-formaldehyde resin, which was stirred and foamed before mixing. Afterward, it was incorporated into the suspension (raw material with phenol-formaldehyde resin) and rested for one hour. Subsequently, the material was transferred onto wax paper in a laboratory heated press, where both the lower and upper pressing plates were heated to a temperature of 130 °C and pressed to a thickness of 10 mm under 2 MPa pressure. The pressing cycle lasted 20 min. After de-loading, the particleboard was moved to a vertical holder to cool evenly and relieve stress until it reached a laboratory temperature of 20 °C. Afterward, the particleboards were cut to the required dimensions of the test specimens and marked with a unique code.

## Analysis of pulp and paper

### Analysis of black liquor

The production of pulp produces a waste product, black liquor. This liquor has been analysed. Its pH was determined potentiometrically, its density was determined pycnometrically, its viscosity was determined viscometrically, its interfacial tension was determined stalagmometrically, and its concentration of alkali lignin was determined using a spectrophotometer.

The pH value of black liquor is determined by the pH of the cooking liquor, which is reduced by the presence of volatile acids in lignocellulosic materials. The viscosity, density, and interfacial tension are essential parameters for thickening the liquor, converting it into cooking liquor, or using it as fuel in paper mills. The alkaline lignin concentration is a value that tells us how much lignin has passed into the liquor from the cooked raw.

### Analysis of pulp

The pulp produced was tested for general properties. These properties include total yield, amount of rejects, and degree of delignification, Kappa number according to ČSN ISO 302^34^.

### Manufacturing of handsheets

The laboratory handsheets were produced on a RAPID-KÖTHEN RK-2A laboratory sheeting machine (Birkenau, Germany) with a basis weight of 80 g/m^2^and subsequently conditioned according to standard conditions for testing mechanical and physical properties according to ISO 5269-2^35^.

### Mechanical properties of handsheets

For both soda and nitrate-alkaline pulp made from the poppy seed, analyses of laboratory handsheets, specifically for tensile properties (ISO 1924-2standard^36^) using equipment from the FRANK-PTI (Birkenau, Germany), burst strength according to ISO 2758:2014^37^using equipment from the FRANK-PTI (Birkenau, Germany), and the Gurley air permeability was also determined using equipment from Lorentzen & Wettre (Stockholm, Sweden) by ISO 5636-5:2013^38^.

## Analysis of particleboard

### Density profile

The determination of the particleboard’s density profile was carried out using the Imal DPX 300-LTE X-ray density profile meter (San Damaso, Italy). This involved deter-mining the distribution of density across the particleboard and its thickness with a precision of 0.05 mm. A sample measuring 50 × 50 mm was placed in the device, with a dividing gauge pad positioned between the bodies and secured using a spring lock to prevent any movement during measurement. The X-ray beam then passes through the profile, and based on the amount of beam that passes through, it determines the highest density at a given point, which is plotted on a graph in kg/m^3^. This is a non-destructive testing meth-od that accurately describes the density distribution within the profile, which affects the mechanical properties of the particleboard. Afterwards, the samples were used for de-lamination testing.

### Internal bonding strength

**Fig. 1:**
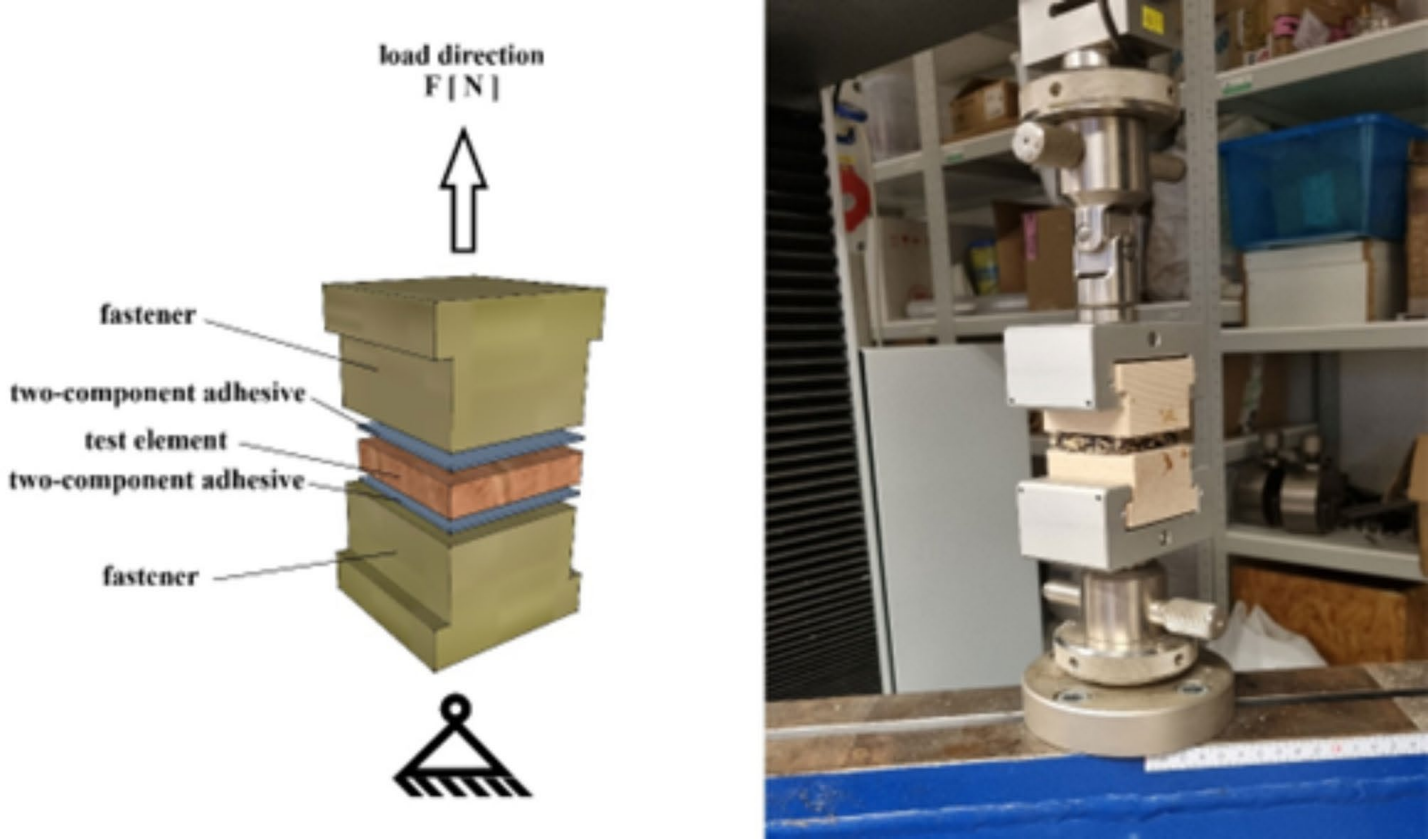
Internal bonding strength testing equipment.

Internal bonding strength was carried out according ČSN EN 311^39^. Test samples were from both sides glued to the T shape beech wood with two-component epoxy adhesive. Dimension of test samples were 50 × 50 mm. For testing was used universal testing device TIRA test 2850 (Schalkau, Germany) test samples were loaded in tensile perpendicular to the plane of a particleboard, Fig. 1.

Internal bonding strength was determined by Eq. 1:

1 $$\:\sigma\:=\frac{{F}_{max}}{a\:.\:b}\:\left[MPa\right],$$

where F_max_ is the maximum tensile force applied to the specimen at the moment of failure (N), and a and b are the length and width of the test specimen (mm).

### Sorption

The determination of sorption was conducted on samples measuring 50 × 50 mm. The initial moisture content of the samples for measurement corresponded to the external conditions of 65% relative humidity and a temperature of 30 °C. Subsequently, the humidity was increased to 95% at a temperature of 30 °C. The samples were weighed at specific time intervals with an accuracy of 0.01 g and recorded along with the elapsed time by ČSN 49 0104^40^. The total duration of the measurement was 75 h. Sorption occurred in a climate chamber throughout the duration of the experiment.

### Water uptake

The determination of the water uptake of samples was carried out using a standardized procedure. Samples measuring 50 × 50 mm with an initial moisture content of approximately 12% were submerged in a container with distilled water by ČSN 49 0104^40^. At predetermined time intervals, the samples were removed, dried, and weighed, then subsequently resubmerged in the water container. This experiment was conducted over a period of 24 h.

## Results

### Chemical analysis

The chemical composition of the plant has a significant effect on fibre properties and pulp yield. Table 1 shows the chemical composition of poppy straw compared to other straw species. The chemical composition of each component may vary according to climate, cultivation, plant part, and other aspects, so these values may differ from other publications on poppy straw processing.

**Table 1:** Chemical composition (in mass % of oven dried samples).

Raw material / Chemical composition	Ash	Extractives	Cellulose	Lignin	Holo-cellulose	Hemi-celluloses
Poppy	1.67 (0.14)	7.53 (0.23)	35.16 (0.65)	22.70 (0.29)	69.70 (1.17)	34.54 (1.57)
Wheat straw^41^	11.63	9.33	50.70	22.33	69.84	19.14
Sugarcane waste^42^	6.45 (0.10)	–	40.04 (0.40)	17.40 (0.20)	73.60 (0.60)	33.20 (0.20)
Rice straw^43^	15.60	–	42.98	16.43	–	–
Rapeseed straw^44^	3.20	3.60	37.70	26.40	–	–
Corn stalks^45^	4.65 (0.20)	–	53.60 (1.70)	20.00 (0.90)	77.50 (1.60)	26.40 (1.20)

* Standard deviation values are in parentheses.

### Analysis of pulp and paper

Among the first analyses in pulp production is the determination of the delignification number, Kappa number. The lower the Kappa number, the lighter the resulting pulp because it is more delignified. The nitrate-alkaline pulp was cooked to a total yield of 37.8% with a delignification number of Kappa 34.19 with a reject content of 0.68%. The soda pulp achieved a higher total yield of 40.3% but also more rejects of 1.03%, and although the pulp was cooked for significantly longer, the Kappa number was only 27.05.

In addition to pulp analysis, the cooking chemical, black liquor, was analysed. In the case of the nitrate-alkaline method, the cooking liquor is taken after extraction with 6% sodium hydroxide when the already nitrated lignin is transferred into the liquor. In the soda method, the black liquor is removed from the cooking vessel before washing the pulp. The values contained in the black liquor are given in Table 2 and are compared with industrial kraft wood pulp.

**Table 2:** The properties of the black liquor.

Pulp / Liquor properties	pH	Density, kg/m^3^	Viscosity, Pa·s	Interfacial tension, mN/m	Concentration of alkaline lignin, g/l
Nitrate-alkaline pulp	12.54 (0.04)	1014.85 (8.31)	1.14 (0.03)	42.74 (2.40)	3.12 (0.01)
Soda pulp	12.72 (0.20)	1036.15 (1.24)	1.22 (0.02)	60.86 (2.70)	6.14 (0.01)
Kraft hardwood pulp^46^	12.20	1071.00	–	–	27.00
Kraft softwood pulp^46^	12.60	1097.00	–	–	56.00

* Standard deviation values are in parentheses.

### Mechanical properties

For both soda and nitrate-alkaline pulp made from poppy seed, analyses of laboratory sheets of approximately 80 g/m^2^ were carried out, specifically for tensile and compressive strengths, and the Gurley air permeability was also determined to compare the properties with particleboards; the measured values are given in Table 3.

**Table 3:** Mechanical properties of poppy paper.

Pulp / Mechanical properties	Breaking length, km	Relative elongation, %	Tensile index, *N*·g/m	Tensile absorption index, J/g	Burst index, kPa	Air perme-ability, s
Nitrate-alkaline pulp	4.69 (0.3)	1.77 (0.01)	45.95 (0.58)	0.79 (0.03)	202.22 (0.44)	8.04 (0.32)
Soda pulp	5.37 (0.05)	2.17 (0.03)	52.71 (0.26)	0.55 (0.04)	234.12 (1.63)	10.66 (0.40)

* Standard deviation values are in parentheses.

### Analysis of particleboards

**Fig. 2:**
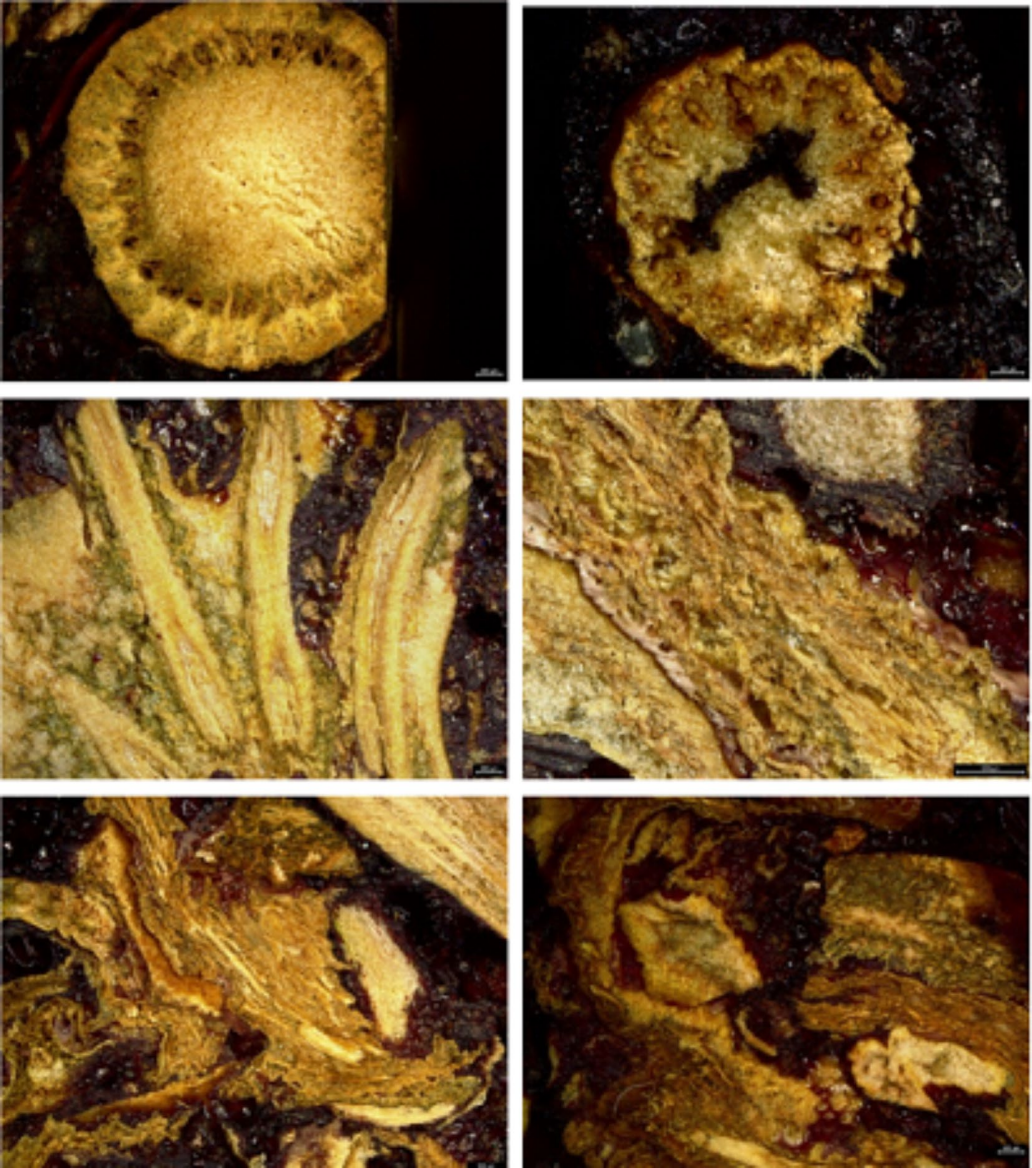
Particleboard structure, resolution 500 μm.

The plates were made in two different thicknesses, which will be compared with each other, namely 10 mm and 8 mm. The structure of the particleboard can be seen in Fig. 2, which was made using a Leica DVM6 M digital microscope (Wetzlar, Germany).

### Density profile

**Fig. 3:**
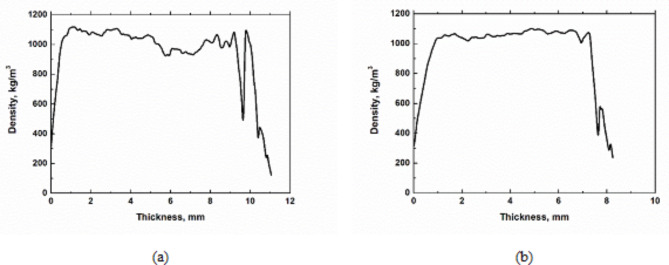
Density profile of the particleboards: (**a**) thickness 11 mm; (**b**) thickness 8 mm.

The density profile data are processed graphically in Fig. 3. Two types of thickness were chosen for different plate applications.

### Internal bonding strength

Table 4 gives the values of the internal bonding strength and the maximum force at the moment of failure in the splitting test.

**Table 4:** Internal bonding strength of poppy particleboard.

Thickness of particleboard	Maximum force, *N*	Internal bonding strength, Mpa
Poppy particleboard, 11 mm	253.88 (9.29)	0.10 (0.02)
Poppy particleboard, 8 mm	561.91 (16.94)	0.22 (0.06)
Waste from paper production with a mixture of wood (60:40), 13 mm^47^	–	0.47 (0.21)

* Standard deviation values are in parentheses.

### Sorption

**Fig. 4:**
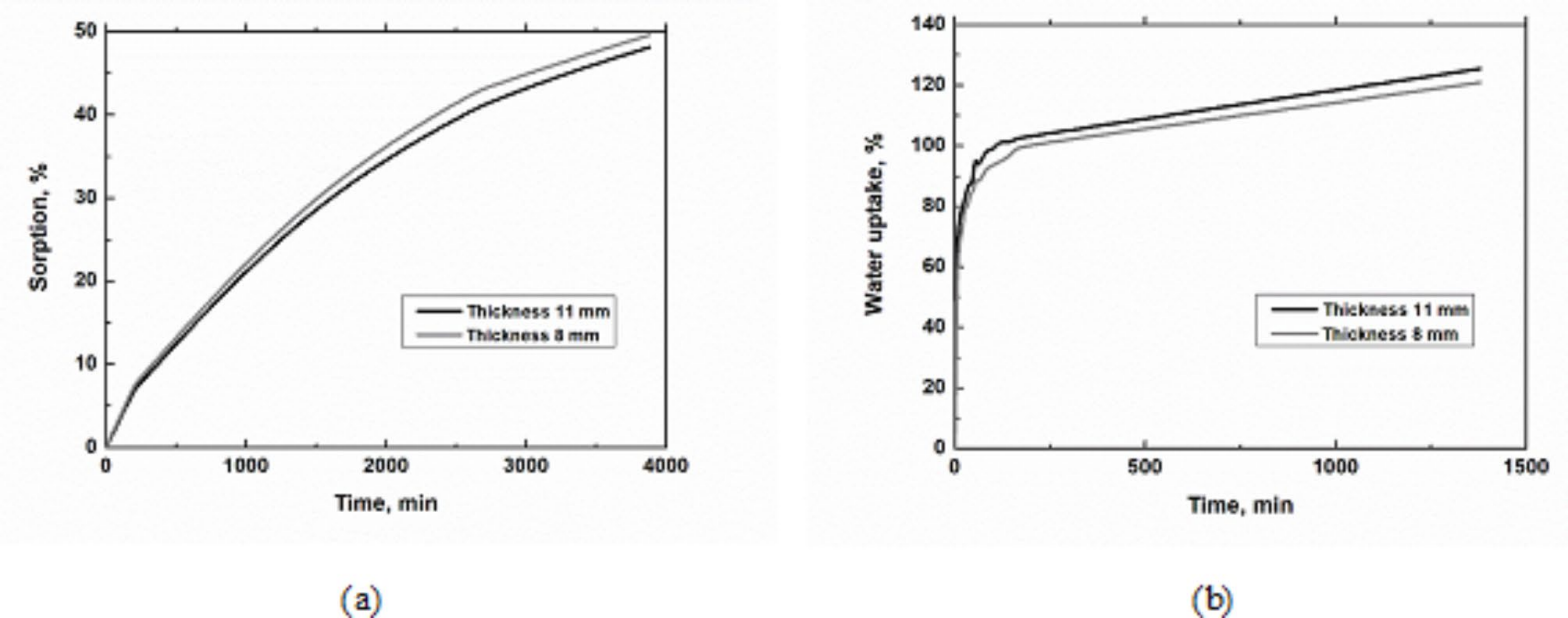
Particleboards: (**a**) Sorption of particleboards; (**b**) Water uptake of particleboards.

Particleboards from each series (thickness 8 and 11 mm) were used to determine the sorption. The process of the determination of sorption is shown in Fig. 4a.

### Water uptake

Similar to the samples for wetness determination, the samples for water absorption determination were measured. The samples at each time interval and their wetting pro-cess are shown in Fig. 4b.

## Discussion

Poppy was compared with other fast-growing raw materials commonly used for paper or particleboard production. Regarding chemical composition, poppy contains significantly less ash than other types of straw, 10% less than wheat straw^41^, and even 14% less than rice straw^43^. In terms of extractives, poppy has almost the same proportion as wheat straw^41^. The Seifert cellulose content is similar to that of rapeseed^44^, but the content was almost 15% higher for wheat^41^and almost 20% higher for corn stalks^45^. However, the cellulose content of 35.2% suggests that poppy straw could be suitable for papermaking, because using raw materials with minimal cellulose content of 30% and with as little ash as possible, which increases the amount of rejects in the pulp. In terms of lignin content, it is higher than sugarcane waste^42^and rice^43^, which could positively affect the products’ mechanical properties. As regards holocellulose, the poppy has about 70% of it, similar to other types of straw. However, its hemicellulose content is higher than that of wheat straw and corn stalks^41,45^; and lower than that of sugarcane waste^42^.

In terms of general pulp properties, the paper was cooked using both methods (soda, nitrate-alkaline) to almost 40% of the total yield, a satisfactory result for a total yield from fast-growing raw materials. For hemp pulp cooked by the kraft and soda process, the yield was at 20%^48^, similar to the soda process for Okra stalks, where, depending on the soda active alkalis, the yield was between 20% and 30%^49^. However, for the neutral sulphite rapeseed semi-pulp, an overall yield of 60 to 70% was achieved, possibly due to the use of less aggressive chemicals. At the same time, the high overall yield showed significantly poorer mechanical properties of the paper^50^. The content of rejects is around 1%, which is very low. Therefore, poppy straws could be suitable for pulp production. In chemical cooking of lignocellulosic material, the yield is usually around 40%, which was achieved in our case. For the kraft technology, which is used for industrial cooking wood pulp, the yield is around 45% maximum, indicating that poppy could be a substitute in wood-scarce countries. Kappa number is the degree of pulp delignification; in our case, it is similar to the kraft technology for conifers, which is around 24.9, and for deciduous trees, 29.9^46^. However, the cooking process is six times longer compared to nitrate-alkaline cooking.

Analysis of the black liquor showed that although the density of the liquor is similar to that of kraft black liquor from wood, the black liquor contains significantly less alkaline lignin. This low concentration of alkaline lignin might be suitable for waste black liquor but unfortunately low for other possible uses of alkaline liquor, such as energy production. However, cooking wood using the kraft method significantly impacts the environment due to the sulphur oxides produced. Nevertheless, air and water purifiers are set up in the paper mills to prevent excessive leakage so that any waste substances produced comply with the European Union regulations. Due to the generation of sulphur oxides, it would be preferable to use soda or nitrate-alkaline technology^46^.

The graphs in Table 3 clearly show that the values for soda pulp are significantly higher than those for nitrate-alkaline poppy straw pulp, both in terms of tensile strength, burst strength, and higher permeability for air.

The breaking length compared to Ates et al.^41^. exceeds that of paper made from agrarian residues by the soda process in both cases. The authors report values of cotton stalk at 3.79 km, tobacco stalk at 4.36 km, reed at 4.18 km, hemp at 3.11 km, and sunflower at 4.24 km.

The relative elongation takes values of 2.17%, close to the results of Bosco et al.^51^. , who focused on sisal waste fibres. In their work, the authors stated that elongation from wood fibres should range from 2% upwards, which is also confirmed by the results – Table 3. However, the relative elongation for nitrate-alkaline pulp is slightly lower; both types are sufficient for production.

Similar to the breaking length, the tensile index of the poppy paper is higher than the tensile strength of the cotton stems at 37.13 N·m/g, the tobacco stems at 42.74 N·m/g, reed at 40.97 N·m/g, hemp at 30.51 N·m/g, sunflower stems 41.61 N·m/g^41^. Both poppy paper types are also satisfactory compared to standard wood pulp paper, whose strength starts at 36 N·m/g^51^. Soda poppy pulp paper also achieves higher values than corn stalks paper 49.1 N·m/g, wheat 41.5 N·m/g^45^, and rice straw 32.84 N·m/g^52^.

Compared to kraft conifer pulp, the tensile strength indices are lower, with unbleached pulp around 70 N·m/g and bleached pulp around 65 N·m/g. However, if the pulp is recycled, the strength decreases with each recycling stage, up to 55 N·m/g for bleached pulp at the third stage. Meanwhile, for chemo-thermo-mechanical cooking, the tensile strength increases with the degree of recycling. For chemo-thermo-mechanical fir pulp from 25 to 40 N·m/g. Therefore, the strength could increase due to recycling in poppy straw cooked by chemo-thermo-mechanical technology (soda and nitrate-alkaline)^53^.

The test handsheets of both the soda pulp 0.79 J/g and the nitrate-alkaline poppy pulp 0.55 J/g achieve values of the tensile absorption index in the same range as the soda pulp from the giant ornamental (*Miscanthus × giganteus*), which range from 0.40 to 0.70 J/g^54^.

The burst strength was almost twice as high as paper made from the agrarian waste of rapeseed 122.4 kPa^50^, cotton 159.1 kPa^41^, sunflower 141.8 kPa^41^, 150–220 kPa for rapeseed neutral sulphite semi-pulp^55^, and wheat 70–180 kPa^56^. At the same time, it reaches almost the same values as pulp carved from rice straw 238.6 kPa, rye straw 244.8 kPa, or barley straw 246.3 kPa^41^. At the same time, however, it is significantly lower than the 510–550 kPa of alfalfa^57^or the 390–416 kPa of switchgrass^58^.

The air permeability, according to Gurley, was similar to that of the corrugated particleboard produced by the kraft process^59^.

We should focus on LCA (life cycle assessment) analysis in the future, as pulp pro-duction has still not been sufficiently studied and analysed. Such analyses are available only for commonly produced pulps made from wood or recovered paper^60–62^. Although Sun et al.^63^. attempted to describe at least a partial analysis of pulp made from wheat straw, bagasse, and bamboo, it would be appropriate to establish a life cycle assessment analysis for poppy straw as well.

The density profile of particleboard tells us how the particleboard was pressed and its strength properties and end-use^64^. The density profile depends on the moisture distribution, the configuration of the particles that enter the press, and the speed and temperature of the pressing or the reactivity of the resin^64^. From the results shown in Fig. 2, the behaviour of the two plate thicknesses is almost identical. The lowest density values were obtained at the edges of the sample, while in contrast, their core reached a density of about 1000 kg/m^3^. The desired density of medium-density particleboards is between 400 and 1000 kg/m^3^, which agrees with our measured values. These values were higher than those for flax shives 588 kg/m^3^ and jute sticks 479 kg/m^3^^65^.

Internal bonding strength is one of the properties investigated in all types of particleboards^66^. Several authors have shown that the tensile strength of a composite material depends on the weight fraction of fibres and their strength. Nevertheless, there is a specific limit, an increase in the proportion of which leads to a decrease in strength^67,68^. Our results were not as good compared to the biomass particleboards by Lee et al.^69^. , but they could have been better. Similar values were achieved, for example, for bagasse glued with polyurethane and castor oil 0.35 MPa^70^, sunflower with urea-formaldehyde resin 0.32 MPa^69^, triticale with low-density polyethylene 0.38 MPa^71^, significantly lower compared to wood-based particleboards glued with melamine-urea-formaldehyde glue 0.72 MPa^72^.

Particleboards made from poppy straw waste achieved approximately 50% water sorption when exposed to standard moisture (Fig. 4a), consistent with most other bio-mass raw materials^69^. The exceptions are peanut husks glued with urea-formaldehyde glue at 98%^69^and particleboards made from jute sticks at 149%^65^.

The water uptake affects the strength and bending stiffness of particleboard. It is influenced by the type of resin and other parameters and substances used in the manufacturing process^66^. In our case, values around 120% were achieved (Fig. 4b), similar to the 93% bagasse and significantly higher than the 35% rice straw^15^.

These sorption tests are performed as basic tests for lignocellulosic products. Moreover, they describe how the boards would behave in a wet environment. They were made in two different thicknesses to describe better how the final product would behave. Another test was the internal bonding strength, which showed properties similar to other fast-growing plants but, unfortunately, significantly lower than wood.

## Conclusion

This research concerned the possible use of waste poppy straw as an alternative source for producing lignocellulosic materials. The suitability of the materials for paper products and particleboard was determined based on the selected properties measured.

While poppy-based particleboards need further optimization to match wood fibres, they show comparable properties to agricultural residues. Future research should focus on improving resin types and pressing conditions to enhance performance.

From the point of view of paper products, poppy pulp produced by the pulping process has better properties than nitrate-alkaline pulp. Compared with other raw materials, waste poppy is an alternative for pulp production, mainly because of its mechanical properties. Because poppy pulp outperforms many agrarian residues in tensile strength and burst index.

Therefore, both applications of waste poppy appear to be alternative raw materials in the future, especially in the case of wood scarcity, which can ensure the continuity of production and composite materials in an economically advantageous and more environmentally friendly way. Using poppy straw has environmental benefits such as reducing deforestation and the carbon footprint, which should position poppy straw as a sustainable alternative as demand for renewable materials increases.

## Data Availability

The datasets used and/or analysed during the current study available from the corresponding author on reasonable request.
